# Evaluation of change in implant abutment after teeth surface modifications

**DOI:** 10.6026/97320630017157

**Published:** 2021-01-31

**Authors:** Khushbu Mishra, Priya singh, Mohammad Kashif Noorani, Kumar Adarsh, Mohammad Nabeel Kalburgi, Manisha Mallik

**Affiliations:** 1 MDS, Consultant periodontist, Patna, Bihar, India; 2 MDS, Consultant prosthodontist & Oral Implantologist, Patna, Bihar, India; 3 Senior Lecturer, Department of Prosthodontics, Dr. B.R. Ambedkar Institute of Dental Sciences & Hospital. Patna, Bihar, India; 4 Senior Lecturer,Department of Orthodontics, Dr. B.R. Ambedkar Institute of Dental Sciences & Hospital. Patna, Bihar, India; 5 Senior lecturer, Department of Prosthodontics, PMNM Dental College and Hospital, Bagalkot, India; 6 Senior lecturer, Department of Periodontology, Buddha institute of dental sciences and hospital, Patna, India

**Keywords:** luting agents, circumferential grooved implant abutments, retentive strength

## Abstract

The surface modifications in teeth increase the retentive strength of cemented castings by providing micro as well as macro retentive ridge and groove patterns. Restoring the dental implants with cement-retained prosthesis is well known. Therefore, it is of interest to compare retentive property of implant abutments with and without circumferential grooves. Hence, 20 straight shoulder type titanium abutments were with abutment screws as well as prefabricated plastic copings and corresponding 12 mm-long stainless steel laboratory implant analogs were used. The abutments were divided into two subgroups of 10 abutments each: without grooves and with grooves. After thermocycling and storing the cemented abutments in water at 37°C water for 6 days they were assembled in the Universal testing machine and subjected to a pullout test (retention) at a crosshead speed of 5.0mm/min to record forces in Newton. Data suggest that the addition of grooves increased the retention. The mean retentive forces of standard machined abutments (plain) cemented with Resin modified GIC showed 339.34 N. Retention increased by 667.39N after addition of circumferential grooves. The surface modification of an implant abutment by means of circumferential grooves is an effective method of improving the retention of cast crowns cemented with resin modified GIC especially in short abutments.

## Background:

The success of oral rehabilitation of dental implants not only depends on osseointegration but also on maintenance of the prosthesis on the implant abutment [1-4]. Implant restorations can be screw retained, cement retained or combination of both [5-8]. The retention of cemented prosthesis has been shown to be influenced by various parameters such as abutment size (height and width), abutment texture, the convergence angle between the walls of the abutment and the cements. Factors that may affect the retention of cast restorations include geometry of abutment preparation, abutment taper, surface area, abutment height, surface roughness, retentive grooves, and the luting agent used [9-11]. Surface roughness, grooves, and luting agents are factors that can be controlled by the clinician [12].

## Material and Method:

20 straight shoulder type titanium abutments were (MIS Implant Technologies Ltd, Misgav, Israel) (6 mm in height with two grooves and 0.5 mm shoulder width) with abutment screws as well as prefabricated plastic copings and corresponding 12 mm-long stainless steel laboratory implant analogs were used (MD-RSM10, MIS Implant Technologies Ltd, Israel). The abutments were divided into two subgroups of 10 abutments each: without grooves and with grooves. Each groove of MIS Implant Tech measured using stereomicroscope 20X magnification was 175.2µm wide and 86.6µm deep. After thermocycling and storing the cemented abutments in water at 37°C water for 6 days they were assembled in the Universal testing machine (computerized, software based, Model No. STS 248) and subjected to a pullout test (retention) at a crosshead speed of 5.0mm/min. The forces required to remove the copings were recorded in Newton.

## Results and Discussion:

**Figure 1:**
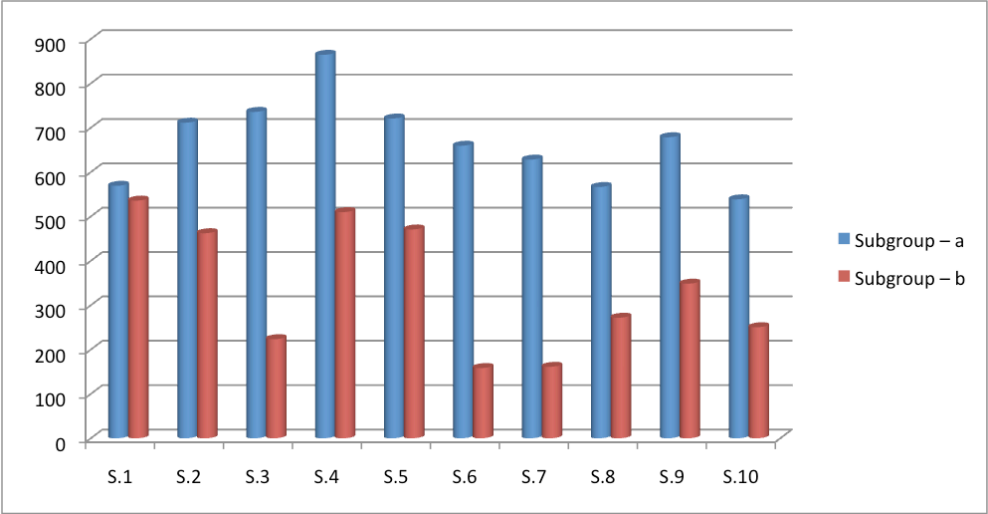
retention of different samples with and without grooves.

The mean tensile force required to separate the castings from the abutments is seen in (Figure 1). It was apparent that the circumferential grooves increased the retention. The one-way ANOVA test indicated that the additional grooves significantly increased the retention of the castings (P<0.05). Cement-retained implant prosthesis have become a method of choice for implant-supported restorations. To increase the retention of these cement retained implant prosthesis, especially in short abutments surface modifications are done by many methods and incorporating circumferential grooves is one of the modification used in this study. Along with this, the selection of appropriate cement is equally important. The null hypothesis that the use of circumferential grooves would not have any effect on the retention of the cemented copings was rejected. The results of the present study show that the use of circumferential grooves increased the retention of the cement-retained copings. Therefore, circumferential grooves can help provide retention control while still maintaining retrievability. The findings of this study suggest that the addition of grooves increased the retention. The mean retentive forces of standard machined abutments (plain) cemented with Resin modified GIC showed 339.34N and after addition of circumferential grooves, retention increased by 667.39N.

The experimental conditions of other studies were not exactly the same. The study done by Lewinstein et al. [12] compared the effect of increasing the number of circumferential grooves on the retention of cemented cast copings on implant abutments. Another study done by Nejatidanesh et al [13] compared the retention values of implant supported metal copings using different luting agents and concluded that the Resin Modified Glass Ionomer, Zinc Phosphate, Zinc Polycarboxylate, and Panavia F2.0 had statistically the same retentive quality and are recommended for definitive cementation of single implant-supported restorations. Walfart et al. [14] investigated the retention of various cements without thermocycling, and found that retentive forces for ZP (Harvard Cement; Harvard Dental International GmbH) was 400N and for ZO (Freegenol; GC Europe NV, Leuven, Belgium) 180N, which are not similar to the current findings as thermocycling reduced the retention values. Clinically, the circumferential grooves can be effective for increasing the retention of fixed dental prostheses in situations where short abutments are used because of small interocclusal distance. The retention test/pullout test was performed and retention values were recorded in Newton. Results proved the circumferential grooves on implant abutments gives better retention when compared with standard machined (plain) abutments. The further scope of present study is that, this protocol did not simulate long-term oral conditions. Therefore, additional studies are needed to quantify the effect of grooves on the retention of other cements under long-term simulation, which may assist clinicians in cement selection. However within the limitations of the study it was concluded that circumferential grooves cemented with Resin modified GIC gives better retention. The results of this in–vitro study can be clinically applied in cases of short abutments by incorporating retentive grooves and Resin modified GIC to enhance the retention of prosthesis.

## Conclusion:

The surface modification of an implant abutment by means of circumferential grooves is an effective method of improving the retention of cast crowns cemented with resin modified GIC specially in short abutments.
